# ATM protein and p53-serine 15 phosphorylation in ataxia-telangiectasia (AT) patients and at heterozygotes

**DOI:** 10.1054/bjoc.2000.1168

**Published:** 2000-05-18

**Authors:** D Delia, S Mizutani, S Panigone, E Tagliabue, E Fontanella, M Asada, T Yamada, Y Taya, S Prudente, S Saviozzi, L Frati, M A Pierotti, L Chessa

**Affiliations:** Department of Experimental Oncology, Istituto Nazionale Tumori, Via G. Venezian 1, Milano, 20133, Italy; Department of Virology, National Children's Medical Research Center, 3-35-31 Taishido, Tokyo, Setagaya-ku, 154, Japan; Biology Division, National Cancer Center Research Institute, Tsukiji 5-1-1, Tokyo, Chuo-Ku, 104, Japan; Department of Experimental Medicine and Pathology, University ‘La Sapienza’, Viale del Policlinico 155, Roma, 00161, Italy; IRCSS Neuromed, Pozzilli, Isernia, Italy; Dept. of Genetics, Biology and Medical Chemistry, University of Torino, Italy

**Keywords:** ataxia-telangiectasia, ATM, cell cycle

## Abstract

ATM (ataxia-telangiectasia mutated) gene plays a central role in the DNA-damage response pathway. We characterized the ATM protein expression in immortalized cells from AT and AT-variant patients, and heterozygotes and correlated it with two ATM-dependent radiation responses, G1 checkpoint arrest and p53-Ser 15 phosphorylation. On Western blots, the full-length ATM protein was detected in eight of 18 AT cases, albeit at 1–32% of the normal levels, whereas a truncated ATM protein was detected in a single case, despite the prevalence among cases of truncation mutations. Of two ataxia without telangiectasia [A-(T)] cases, one expressed 20% and the other ~70% of the normal ATM levels. Noteworthy, among ten asymptomatic heterozygous carriers for AT, normal amounts of ATM protein were found in one and reduced by 40–50% in the remaining cases. The radiation-induced phosphorylation of p53 protein at serine 15, largely mediated by ATM kinase, was defective in AT, A(-T) and in 2/4 heterozygous carriers, while the G1 cell cycle checkpoint was disrupted in all AT and A(-T) cases, and in 3/10 AT heterozygotes. Altogether, our study shows that AT and A(-T) cases bearing truncation mutations of the ATM gene can produce modest amounts of full-length (and only rarely truncated) ATM protein. However, this limited expression of ATM protein provides no benefit regarding the ATM-dependent responses related to G1 arrest and p53-ser15 phosphorylation. Our study additionally shows that the majority of AT heterozygotes express almost halved levels of ATM protein, sufficient in most cases to normally regulate the ATM-dependent DNA damage-response pathway. © 2000 Cancer Research Campaign


*British Journal of Cancer* (2000) **82**(12), 1938–1945
© 2000 Cancer Research Campaign
doi: 10.1054/ bjoc.2000.1168, available online at http://www.idealibrary.com on IDEAL®

### ATM protein and p53-serine 15 phosphorylation in ataxia-telangiectasia (AT) patients and at heterozygotes

**D Delia[1], S Mizutani[2], S Panigone[1], E Tagliabue[1], E Fontanella[1], M Asada[2], T Yamada[2], Y Taya[3], S Prudente[4], S Saviozzi[6], L Frati[4,5], MA Pierotti[1] and L Chessa[4]**

[1]Department of Experimental Oncology, Istituto Nazionale Tumori, Via G. Venezian 1, 20133 Milano, Italy; [2]Department of Virology, National Children's Medical Research Center, 3-35-31 Taishido, Setagaya-ku, Tokyo 154, Japan; [3]Biology Division, National Cancer Center Research Institute, Tsukiji 5-1-1, Chuo-Ku, Tokyo 104, Japan; [4]Department of Experimental Medicine and Pathology, University 'La Sapienza', Viale del Policlinico 155, 00161 Roma; Italy; [5]IRCSS Neuromed, Pozzilli, Isernia, Italy; [6]Dept. of Genetics, Biology and Medical Chemistry, University of Torino, Italy

**Summary** ATM (ataxia-telangiectasia mutated) gene plays a central role in the DNA-damage response pathway. We characterized the ATM protein expression in immortalized cells from AT and AT-variant patients, and heterozygotes and correlated it with two ATM-dependent radiation responses, G1 checkpoint arrest and p53-Ser 15 phosphorylation. On Western blots, the full-length ATM protein was detected in eight of 18 AT cases, albeit at 1–32% of the normal levels, whereas a truncated ATM protein was detected in a single case, despite the prevalence among cases of truncation mutations. Of two ataxia without telangiectasia [A-(T)] cases, one expressed 20% and the other ~70% of the normal ATM levels. Noteworthy, among ten asymptomatic heterozygous carriers for AT, normal amounts of ATM protein were found in one and reduced by 40–50% in the remaining cases. The radiation-induced phosphorylation of p53 protein at serine 15, largely mediated by ATM kinase, was defective in AT, A(-T) and in 2/4 heterozygous carriers, while the G1 cell cycle checkpoint was disrupted in all AT and A(-T) cases, and in 3/10 AT heterozygotes. Altogether, our study shows that AT and A(-T) cases bearing truncation mutations of the ATM gene can produce modest amounts of full-length (and only rarely truncated) ATM protein. However, this limited expression of ATM protein provides no benefit regarding the ATM-dependent responses related to G1 arrest and p53-ser15 phosphorylation. Our study additionally shows that the majority of AT heterozygotes express almost halved levels of ATM protein, sufficient in most cases to normally regulate the ATM-dependent DNA damage-response pathway. © 2000 Cancer Research Campaign

**Keywords**: ataxia-telangiectasia; ATM; cell cycle

Ataxia telangiectasia (AT) is a rare autosomal recessive multi-systemic disorder associated with an elevated risk of malignancy, primarily leukaemias and lymphomas (Boder, 1985). It is estimated that up to 1% of the general population are heterozygous carriers of AT mutations and, albeit asymptomatic, these individuals have an increased predisposition to cancer in adulthood (Swift et al, 1991).

The gene responsible, ATM (ataxia telangiectasia mutated), encodes a nuclear 350 kDa phosphoprotein (Savitsky et al, 1995*a*, 1995*b*) containing a carboxy terminus phosphatidylinositol 3-kinase (Pl-3 kinase) catalytic domain shared by members of a superfamily of large eukaryotic proteins involved in intracellular signalling, DNA-damage induced cell cycle checkpoints, DNA repair and recombination (Meyn, 1995; Hoekstra, 1997). In the DNA-damage response pathway, ATM acts upstream of p53 to induce cell cycle arrest at the G1/S and G2/M boundaries and a slowing of the S-phase (Hoekstra, 1997). Signalling by ATM involves interactions with and phosphorylation of critical molecules, including the mitotic checkpoints Chk1 and Chk2 (Sanchez et al, 1997; Walworth et al, 1997), and p53 (Watters et al, 1997; Westphal et al, 1997; Banin et al, 1998; Canman et al, 1998).

*Received 16 July 1999*
*Revised 17 December 1999*
*Accepted 16 February 2000*

*Correspondence to:* D Delia

AT patients are affected by a wide spectrum of homozygous or compound heterozygous germ line mutations of the ATM gene, primarily deletions or insertions that inactivate the ATM protein (Savitsky et al, 1995*a*; Gilad et al, 1996; Lakin et al, 1996; Wright et al, 1996). ATM gene mutations have also been found in T-cell prolymphocytic leukaemia patients with no family history of AT and in non-Hodgkin's lymphomas (Vorechovsky et al, 1997), but in contrast to AT, these mutations are somatic, mostly missense and frequently associated with loss of the normal allele.

The abnormal responses of AT cells to ionizing radiation include increased chromosomal breaks and radioresistant DNA synthesis (Stilgenbauer et al, 1997), impaired p53-dependent arrest in G1 and other cell cycle checkpoint defects (Canman et al, 1994; Thacker 1994; Takagi et al, 1998) that can be corrected by ectopic expression of a functional ATM protein (Khanna et al, 1995; Ziv et al, 1997). Cultured fibroblasts and lymphocytes from AT heterozygotes exhibit chromosomal radiosensitivity values intermediate between normal and AT (West et al, 1995; Scott et al, 1996; Shigeta et al, 1999), indicating a partial impairment of the ATM-dependent DNA damage-response pathway and suggesting a link between increased incidence of cancer and dysfunctional response to low-dose radiation exposure among AT heterozygotes.

We have produced anti-ATM antibodies to analyse the relationship between ATM protein expression and regulation of the ATM-dependent G1 cell cycle checkpoint and p53-Ser 15 phosphorylation in AT patients and heterozygotes. We report that the normal size ATM protein is either absent or poorly expressed in AT and atoxia without telangiectasia [A(-T)] whereas the truncated ATM protein is rarely detectable. Interestingly, compared to normal controls, most AT heterozygotes exhibit reduced levels of ATM protein. We also show that p53-Ser15 phosphorylation, largely mediated by the kinase activity of ATM, is markedly reduced in AT and A(-T), even in cases with detectable ATM protein. Finally, cell cycle analysis 24 h post-irradiation show a G1 checkpoint defect in all AT, A(-T) and in some heterozygotes.

### MATERIALS AND METHODS

### Cell lines, irradiation and cell cycle analysis

Peripheral blood mononuclear cells (PBMC) from normal donors, AT patients and relatives were immortalized with the supernatant of the Epstein–Barr virus (EBV)-producing cell line B95-8 and grown at 37°C in 5% carbon dioxide in RPMI-1640 (Bio-Wittaker, Walkersville, MD, USA) plus 15% heat-inactivated fetal calf serum, 100 U ml$^{-1}$ penicillin, 100 μg ml$^{-1}$ streptomycin, 2 mmol l$^{-1}$ glutamine. PBMC were activated by treatment for 96 h with 1 nM TPA (Sigma, St Louis, MO, USA) and 0.5 μg ml$^{-1}$ ionomycin (Calbiochem, San Diego, CA, USA) as described (Delia et al, 1984). Irradiations were performed by an IBL437CO instrument with a $^{137}$Cesium source providing 798 cGy min$^{-1}$. Control samples were held at room temperature for the duration of the irradiation. For cell cycle analysis, samples were resuspended in phosphate-buffered saline (PBS) plus 0.1% saponine and 1 μg ml$^{-1}$ RNAase (Sigma), incubated for 20 min at 37°C, stained with 25 μg ml$^{-1}$ propidium iodide (Sigma) and analysed (20 000 cells USA per histogram) by flow cytofluorimetry (FACSvantage, Becton-Dickinson, Palo Alto, CA, USA), as reported (Delia et al, 1997).

### Analysis of ATM gene mutations

Mutations were identified by restriction endonuclease fingerprint (REF) (Gilad et al, 1998) or protein truncation test (PTT). Segregation analysis on AT relatives were performed by means of reverse transcription PCR (RT-PCR), restriction and Heteroduplex analyses in order to identify the heterozygotes (Prudente et al, 1998). The quantification, in some cases, of the mutant and wild-type ATM alleles was performed by RT-PCR using primers flanking the ATM mutation and primers for gluceraldehyde 3-phosphate dehydrogenase (GAPDH) for normalization.

### Recombinant protein, antibody preparation, SDS-PAGE, immunoblot analysis

The cDNA sequence of ATM corresponding to amino acids 1–150, amplified by RT-PCR from mRNA derived from normal cells, was cloned in-frame with a 6 × Hist-tag sequence in the expression vector pTrc-His-B (Invitrogen, San Diego, CA, USA). After transformation with the resulting pTrc-His-ATM-N plasmid and 6 h induction with 100 mM isopropylthio-(-D-galactoside), bacteria were lysed in 6 M urea, sonicated, centrifuged and the recombinant protein was purified from the supernatant by a TALON column (Clontech, Palo Alto, CA, USA) and 20–100 mM imidazole elution. Sera from rabbits and mice immunized with the recombinant protein were affinity-purified. Cell extracts were prepared as described (Delia et al, 1997), by lysing 5 × 10$^6$ cells in 200 μl buffer containing 125 mM Tris–HCl pH 6.8, 5% sodium dodecyl sulphate (SDS). After boiling for 2 min and addition of the inhibitors 1 mM phenylmethyl sulphexyl fluoride (PMSF), 10 μg ml$^{-1}$ pepstatin, 100 KIU ml$^{-1}$ aprotinin, 10 μg ml$^{-1}$ leupeptin (all from Calbiochem) and 1 mM sodium orthovandate (Sigma), lysates were sonicated for 20 s, centrifuged, quantitated by the micro-BCA method (Pierce, Rockford, IL, USA) and fractionated (100 μg per lane) on a two-gradient SDS polyacrylamide gel electrophoresis (SDS-PAGE) (bottom 4 cm: 10% gel, acrylamide/bis-acrylamide ratio 29:1; top 6 cm: 5% gel, acrylamide/bis-acrylamide ratio 100:1) that resolved on the same gel both β-actin (MW 45 kDa) and ATM (MW 350 kDa). Molecular weight rainbow standards were from Amersham. Gels were electroblotted overnight onto polyvinylidene difluoride (PVDF) membranes (Millipore, Bedfored, MA, USA) in 25 mM Tris, 192 mM Glycine pH 8, 20% methanol at 300–400 mA. After blocking with 5% non-fat dried milk in PBS plus 0.1% Tween-20 (PBS-T) (Sigma), membranes were cut into two halves by the 220 kDa marker, and incubated for 3 h with the anti-ATM antibody (upper half) and the anti β-actin (Sigma) (lower half) diluted in TBS-T plus 5% milk. After rinsing in PBS-T and incubation for 1 h with 1:3000 dilution of peroxidase-labelled secondary antibody, membranes were rinsed again and revealed by ECL-plus (Amersham) and autoradiography. Western analysis for p53 were performed on lysates from cells harvested before and 3 h after 400 rads γ-radiation, and separated on 12% SDS-PAGE. The phosphorylation of p53 at serine 15 was detected using a phosphospecific rabbit antibody (Shieh et al, 1997), and total p53 using the monoclonal antibody DO7. Autoradiographs were scanned and quantitated using ImageQuant (Molecular Dynamics, Sunnyvale, CA, USA).

### RESULTS

### ATM protein levels in AT patients and AT heterozygotes

Eighteen AT cases were included in this study, five homozygous (AT9RM, AT21RM, AT32RM, AT43RM, AT44RM and AT74RM) and nine compound heterozygous (AT15RM, AT35RM, AT52RM, AT57RM, AT58RM, AT65RM, ATJ1 and the siblings AT28RM and AT54RM) for ATM gene mutations predicted to result in protein truncation in all but AT21RM, the latter bearing a 90 nt deletion mutation causing exon 18 skipping (Table 1). Mutations in the remaining cases are currently unknown. On Western blots (Table 1 and Figure 1), ten cases were negative, whereas seven others evidenced a normal size ATM protein band whose intensity was 1 ÷ 32% of normal cells. A truncated ATM product of ~270 kDa was detected in ATJ3 (Figure 1, bottom), a case with an unknown gene mutation. Low amounts of ATM protein were observed in A(-T) 6RM (20%), but not in A(-T)5RM (70%). Among AT heterozygotes, ATM gene mutations were established in eight of ten, all bearing one normal and a second mutated allele (Table 1). Some of them are obligate heterozygotes, e.g. 154RM and 155RM are the father and mother respectively, of AT28RM and AT54RM siblings, while 227RM and 247RM are fathers of AT43RM and AT44RM respectively. All cases, except 98RM, expressed lower amounts of ATM protein than normal controls (e.g. LBC-N and cells from healthy members of AT families with no ATM mutations such as 242RM and 243RM, both uncles of AT44RM; and 314RM uncle of another AT

© 2000 Cancer Research Campaign

**Table 1** ATM genomic mutations, protein levels and cell cycle analysis in cells from AT patients, AT-heterozygotes and normal individuals

| Case | ATM genomic mutations | ATM protein levels[c] | G1/G2-M[e] |
|---|---|---|---|
| AT9RM | 9139 C→T | 0.07 | 0.34±0.064 (3) |
| AT15RM | 129T→C / ? | 0.02 | 0.8 |
| AT21RM | IVS18 + 1 del G | 0.32 | 0.88±0.29 (3) |
| AT27RM | NA | <0.03 | 0.69±0.17 (3) |
| AT28RM[b] | 7792 C→T / 8283 del TC | neg | 1.04±0.33 (4) |
| AT32RM | 3802 del G | <0.03 | ND |
| AT35RM | 3576 G→A / 5574 G→A | <0.02 | 0.83±0.16 (3) |
| AT43RM | 7517 del 4 | Neg | 0.77±0.41 (3) |
| AT44RM | IVS 12 + 1 G→T | Neg | 0.6 |
| AT52RM | 7327 C→T / 8365 del A | Neg | 0.93±0.23 (4) |
| AT54RM[b] | 7792 C→T / 8283 del TC | Neg | 0.95±0.07 (3) |
| AT57RM | 7517 del 4 / ? | Neg | 0.33±0.07 (3) |
| AT58RM | 2250 G→A / ? | Neg | ND |
| AT65RM | IVS47 –9 G→A / 8814 del 11 | Neg | 0.61±0.13 (3) |
| AT68RM | NA | Neg | 1 |
| AT74RM | [3993ins29][a] | 0.15±0.02 | ND |
| ATJ1 | 7519 del GA / ? | <0.02 | ND |
| ATJ3 | NA | 0.29[d] | 0.7 |
| A(-T)5RM | NA | 0.7±0.1 | 0.66±0.15 (3) |
| A(-T)6RM | NA | 0.20 | 1.15±0.1 (4) |
| 75RM | 3894ins T / N | 0.53 | 1.75±0.2 (3) |
| 98RM | IVS 18 + 1 del G / N | 0.9±0.1 | 0.99±0.15 (4) |
| 154RM | 8283 del TC / N | 0.5 | ND |
| 155RM | 7792 C→T / N | 0.45±0.18 | 1.67±0.09 (3) |
| 227RM | 7517 del 4 / N | 0.40±0.14 | 1.12±0.17 (4) |
| 247RM | IVS12 + 1 G→T / N | 0.45±0.31 | 1.88±0.40 (4) |
| 373RM | 8283 del TC / N | 0.59±0.4 | 2.1±0.24 (3) |
| 261RM | NA | 0.57±0.13 | 2.15±0.63 (3) |
| 262RM | 3802 del G / N | 0.52 | 0.88±0.16 (5) |
| 313RM | NA | 0.35±0.28 | 1.9 |
| 242RM | N | 1.01±0.07 | 2.1±1.0 (5) |
| 243RM | N | 1 | 1.57±0.81 (4) |
| 314RM | N | 1.2±0.2 | 2.7±0.59 (3) |
| LBC-N | N | 1 | 2.18±0.53 (9) |

N, normal allele or normal case; NA, mutation not available or not yet identified. [a]Mutation position referred to the cDNA; [b]sibs; [c] the ATM intensity values were calculated from densitometric analysis of Western blot autoradiographic films, after correction for protein content using β-actin as a marker (see Materials and Methods) and normalization against normal LBC-N cells. The average intensity ± s.d. has been determined on cases analysed 3 or more independent times. [d]Value referred to the truncated ATM protein. [e]DNA flow cytofluorimetric analysis were performed on propidium iodide stained cells 24 h after exposure (or not, for control) to 400 rads of γ-radiation. The fractions of G1 and G2-M cells were determined by CellFIT software analysis of the acquired DNA histograms (20 000 events/sample). For cases analysed multiple times, the G1/G2-M ratio (average ± s.d.) is shown together with the number of independent measurements (in parenthesis).

case). In 98RM (father of AT21RM) bearing a mutation causing exon 18 skipping, the ATM protein was just below normal levels. Reduced ATM expression was found in two relatives of AT patients with yet unknown mutations (261RM and 313RM), suggesting the possibility that these cases are heterozygous for AT.

To rule out the possibility that the differences in ATM expression between normal and AT heterozygotes were somehow due to EBV immortalization, protein determinations were performed on in vitro activated T-cells from parents of two AT cases. It can be seen (Figure 2) that activated T-cells from the AT heterozygotes 154RM and 155RM exhibited 50% lower levels of ATM protein than activated T-cells from normal donors.

### Phosphorylation of p53 at serine 15

In normal cells, p53 protein becomes rapidly phosphorylated at serine 15 following DNA damage, whereas in AT cells this event is defective (Shieh et al, 1997; Siliciano et al, 1997; Nakagawa et al, 1999). This post-translational modification of p53 is primarily mediated by ATM, whose kinase activity is markedly enhanced by treatment with radiomimetic drug (Banin et al, 1998; Canman et al, 1998). We thus examined the phosphorylation of p53-Ser 15 to evaluate the activity of ATM in relation to ATM gene mutations and protein levels. In all cases, unirradiated samples generated a very faint band on immunoblots probed with an antibody for phosphoserine 15 of p53 (Shieh et al, 1997). However, at 3 h post 400 rads γ-radiation, the phosphorylation band was rather intense, but according to densitometric analysis the signal intensity in AT cells was ~60% lower than in normal cells (Figure 3 and 4), irrespective of the amount of endogenous ATM protein (e.g. compare AT65RM with AT21RM). Abnormal p53-Ser15 phosphorylation levels were also found in A(-T)5RM, raising the possibility that the kinase activity of the ATM protein expressed by this case (65% of the normal levels) is impaired. Among four heterozygotes,

 *© 2000 Cancer Research Campaign*

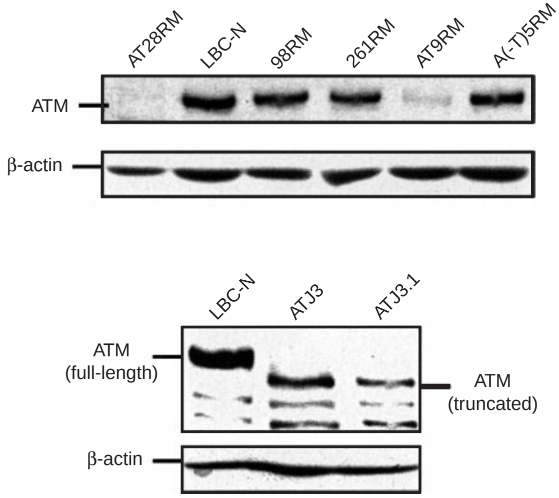

**Figure 1** ATM protein levels in lymphoblastoid cells from normal donors, AT patients and carriers. Western blots were performed as detailed in Materials and Methods, using a two-gradient SDS-PAGE in order to recover on the same membrane both ATM and β-actin. After electroblotting, the upper part of the membrane was tested for ATM and the lower part for β-actin. Binding of antibodies to the membrane was detected by ECL. ATJ3.1 (bottom figure) is the brother of ATJ3

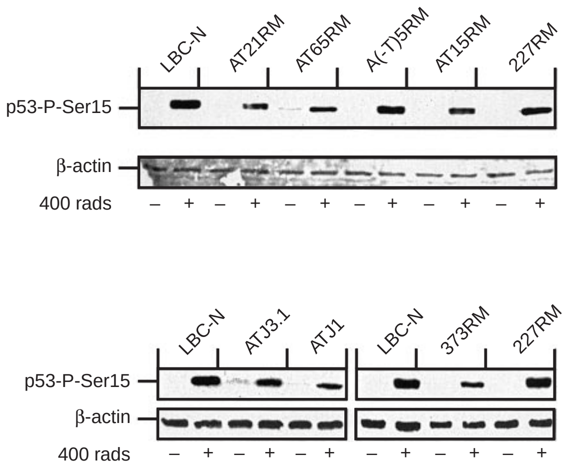

**Figure 3** Radiation-induced phosphorylation of p53 on Serine 15 in AT, A(-T) and heterozygotes. Cells were harvested 3 h after treatment (or not, for control) with 400 rads of γ-radiation, lysed and analysed for p53-Ser 15 phosphorylation by Western blot using a phosphospecific rabbit antibody (see Materials and Methods) and ECL detection. Blots were reprobed for β-actin for normalization

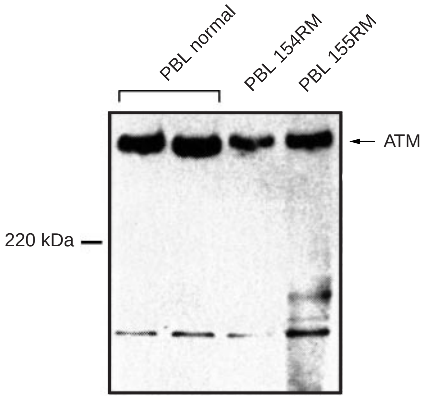

**Figure 2** ATM protein in activated blood lymphocytes. PBMC from normal donors and AT heterozygotes were activated in vitro with TPA-ionomycin for 4 days, thereafter lysed and analysed for ATM by Western blotting p53-Ser15 phosphorylation levels were normal in 98RM and 247RM, but reduced by 20 and 60% in 227RM and 373RM respectively. Of these cases, the latter three expressed reduced amounts of ATM protein. It should be noticed that the partial phosphorylation of p53-Ser15 in ATM negative cases most likely reflects the activity of the AT- and Rad-3-related ATR kinase, reported to phosphorylate this residue, though less efficiently than ATM (Canman et al, 1998; Lakin et al, 1999).

We also performed, in some cases, time-course analysis to determine if the phosphorylation of p53-Ser15 in cells with inactive ATM was somehow delayed. The results (Figure 5) showed that in LBC-N normal cells p53-Ser15 phosphorylation levels remained maximal and stable between 3 and 6 h post irradiation, to decline 10 h later by ~50%. Conversely, in ATJ3 p53-Ser15 phosphorylation progressively increased and by 10 h its levels were comparable to those seen in normal cells at 3 h post-treatment. In A(-T)5RM the trend of p53-Ser15 phosphorylation was similar to that of normal cells, except that at all time-points the signal intensity was reduced.

### Cell cycle analysis

In response to DNA damage, whereas normal cells arrest in G1, cells from AT cells patients do not because of a G1 cell cycle checkpoint defect. This abnormality, associated with an insufficient accumulation of p53 protein and poor induction of its target gene p21$^{waf1}$ (Canman et al, 1994; Khanna et al, 1995) can be corrected by enforced expression of functional ATM (Zhang et al, 1997; Ziv et al, 1997).

We analysed the flow cytofluorimetric DNA profile of cells before and 24 h after treatment with 400 rads of γ-radiation to evaluate the G1 checkpoint function. In all AT and A(-T) cases, irradiation caused a significant reduction of the fraction of G1 and a concomitant increase of G2-M phase cells, and therefore an inversion of the G1/G2-M ratio (Table 1), in marked contrast to the modest redistribution of these cell cycle phases in normal cells (G1/G2-M ratio: 2.18 ± 0.53). Of note, in AT samples the magnitude of the G1 defect was similar, whether or not they expressed ATM protein, suggesting that protein is ineffective. Among eight AT heterozygotes, the G1 checkpoint was abnormal in 98RM, 262RM and 227RM.

Altogether, these evidences lead to conclude the G1 checkpoint is invariably disrupted in AT and A(-T) cases, and this defect is not alleviated by the expression of limiting amounts of ATM protein. In AT heterozygotes, the G1 checkpoint is less frequently abnormal (three of eight cases examined), but no clearcut correlation with the type of ATM mutations has been established.

*© 2000 Cancer Research Campaign* 

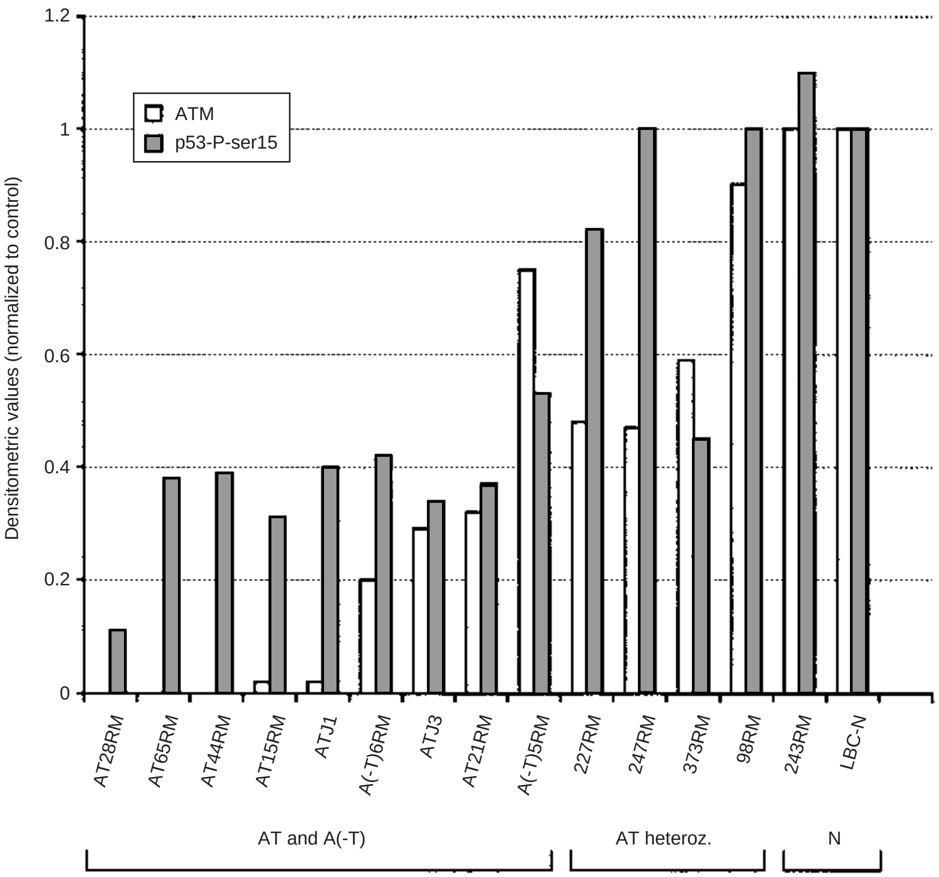

**Figure 4** Levels of phosphorylated p53-Ser15 in AT, A(-T) and heterozygotes in relation to ATM protein. The phosphorylation of p53-ser15 was detected by Western analysis (see legend to Figure 3), and quantitated by scanning densitometry of autoradiographic films. The p53-Ser15 values were corrected for β-actin content, and normalized to those of LBC-N normal cells. The ATM values are those reported in Table 1

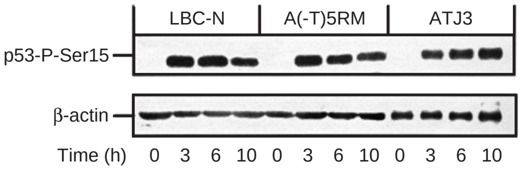

**Figure 5** Time-dependent phosphorylation of p53-Ser15 in response to DNA damage. Cells, unirradiated or harvested at the indicated time-points after 400 rads γ-radiation, and analysed by Western blotting with antibodies to p53 phosphorylated on Ser 15 and to β-actin

### DISCUSSION

The identification of the ATM gene (Savitsky et al, 1995*a*, 1995*b*) is providing new insights for the comprehension of the molecular events that account for the AT phenotype and the role of this gene in genomic instability and cancer predisposition. ATM is believed to be the most common cancer susceptibility gene, since ~ 1% of the general population are heterozygous carriers (Swift et al, 1986; Tchirkov et al, 1997), and these people are at increased risk of cancer (Swift et al, 1991). The suggested relationship between AT heterozygosity and predisposition to early onset of breast cancer remains debated (Swift et al, 1986; Athma et al, 1996; FitzGerald et al, 1997; Janin et al, 1999).

In this study we analysed the ATM expression in lymphoblastoid cells derived from healthy and affected members of AT families in relation to the ATM gene mutations. Variable amounts of full-length ATM protein were found among AT, with ten cases negative and eight showing 1–32% of normal. However, none of the cases, except ATJ3, evidenced truncated ATM proteins despite the prevalence of ATM gene mutations expected to yield polypeptides truncated downstream of the epitope recognized by the anti-ATM antibody in this regard, our data agree with others showing minimal or no ATM protein expression in AT cells affected by different truncation mutations (Lakin et al, 1996; Watters et al, 1997; Gilad et al, 1998; Teraoka et al, 1999). We cannot currently exclude that the lack of protein may reflect a truncation-related instability that affects both the ATM transcript and protein product, according to RT-PCR analysis on AT44RM (and its family members), in which the mutant mRNA accumulates to 60% of the normal levels (data not shown), even though no ATM protein is detected.

ATJ3 (and its affected family member ATJ3.1) was the only case showing a truncated protein, suggesting that the as yet unknown ATM mutation of this case might have milder effect on the destabilization of the transcript and/or protein.

*© 2000 Cancer Research Campaign*

Our data showing modest amounts of full-length ATM protein in cases bearing truncation mutations are concordant with those of Gilad et al (1998) and have been previously explained by the 'leakiness' of these mutations, therefore allowing expression of low levels of ATM protein (Gilad et al, 1998). The residual expression of ATM protein in some AT patients appears associated with a milder disease progression (Gilad et al, 1998).

We have shown that the ATM protein content in AT heterozygotes is ~40–50% lower than normal controls, in agreement with our previous report based on a smaller number of cases (Shigeta et al, 1999). A reasonable explanation for this haplo-insufficiency may lie in the fact that truncation mutation alleles are poorly expressed (as in AT), and thus the total amount of ATM protein in AT heterozygotes mostly accounts for by the expression of the wild-type allele. ATM protein deficit was also found in activated blood T-lymphocytes from the obligate heterozygotes 154RM and 155RM, strongly suggesting that, at least in these cases, the differencies in ATM expression among EBV-immortalized lymphoblastoid cells are real. However, we cannot exclude a certain cell-to-cell line variable influence of EBV on ATM that could account for the observed wide range of protein expression.

The evidence that AT heterozygotes are more radiosensitive than normal donors (West et al, 1995; Scott et al, 1996; Tchirkov et al, 1997; Shigeta et al, 1999) has led to suggestions that the increased risk of cancer in these individuals arises from a partial defect of the gene responsible for AT. Since ATM plays a central role in cell cycle checkpoint responses to DNA damage (Canman et al, 1994; Khanna et al, 1995), we determined the G1 checkpoint function in relation also to ATM protein levels. None of the AT and A(-T) cases analysed arrested in G1 after irradiation, and furthermore, the magnitude of this abnormality did not correlate with the levels of endogenous ATM protein (compare cases negative, e.g. AT57RM, AT54RM, and positive, e.g. AT9RM, AT21RM for ATM protein). This finding would suggest that either the amount of ATM protein in these cases is insufficient to exert a phenotypic effect (in which case A(-T)5RM would represent an exception as it shows 70% of the normal levels of ATM but a defective G1 checkpoint), or that the ATM protein is intrinsically inactive. Three of eight AT heterozygous cases showed an abnormal G1 checkpoint, two of them with ~50% (227RM and 262RM) and one (98RM) with almost normal levels of ATM protein. Interestingly, 98RM is the father of AT21RM, the latter bearing a homozygous mutation causing exon 18 deletion and expressing intermediate levels of ATM (~32% of normal). Whether in this case the mutant protein exerts a dominant negative effect over the wild-type allele, as in the case ATM proteins mutant in the leucine zipper motif (Morgan et al, 1997), is a possibility that requires further investigation.

The fact that many AT carriers exhibit a normal G1 checkpoint, in spite of the reduced ATM expression, suggests that in heterozygosity this level of endogenous protein is still able to provide a normal regulation of the ATM- and p53-dependent G1 cell cycle checkpoint. We cannot, however, exclude that ATM haplo-insufficiency may affect the S phase checkpoint responsible for the intermediate radiosensitivity in AT heterozyygotes (West et al, 1995; Scott et al, 1996; Tchirkov et al, 1997).

In response to DNA damage, p53 undergoes phosphorylation at multiple sites, including serine 15. The phosphorylation of this residue alleviates the binding of p53 to MDM2 and allows p53 itself to accumulate and to exert its function (Shieh et al, 1997). p53-Ser15 phosphorylation is mediated in vitro and in vivo, by the kinases ATM and ATR, whose activities are enhanced by treatment of cells with DNA damaging agents (Banin et al, 1998; Canman et al, 1998; Lakin et al, 1999). It should be noticed however, that while ATM acts immediately after DNA damage, ATR acts at a later time and less efficiently that ATM. We have shown here that although DNA damage induced p53-Ser15 phosphorylation in AT and A(-T) cells, nevertheless, compared to normal cells, the magnitude of this event at 3 h was reduced by ~60% even in cases bearing some ATM protein (e.g. AT65RM vs AT21RM). Two conclusions can be drawn from these results. The first, based on the partial phosphorylation p53-Ser 15 in ATM null cells, which further supports the role in vivo of an additional kinase, most likely ATR (Canman et al, 1998; Lakin et al, 1999; Tibbets et al, 1999), in the phosphorylation of p53. The second, that the ATM protein in AT or A(-T) cases is dysfunctional since its presence was not associated with an increased p53-Ser 15 phosphorylation. The p53-Ser15 phosphorylation in four heterozygotes (three of them showing diminished ATM expression) have shown normal regulation in two, but reduced by 20% and 60% in the other two cases, suggesting that in heterozygosity (wt/mut) the ATM protein can provide a normal function even if present at half the normal levels (e.g. 247RM), although in some cases and for unknown reasons, this may not be so (e.g. 373RM). It is worthnoting that discrepancies between p53-Ser15 phosphorylation and G1 checkpoint control have been found in some cases (e.g. 373RM, 98RM and 227RM, the former exhibiting low Ser 15 phosphorylation and normal G1 arrest, and vice versa for the latter two). Currently we have no explanation for this and we can only hypothesize, in the case of 98RM and 227RM the existence of additional abnormalities in the p53-response pathway that can contribute to these findings.

The analysis of p53 regulation after DNA damage have shown interesting differences in the kinetics of p53-Ser 15 phosphorylation in AT cells. In fact, while in normal cells this phosphorylation becomes maximal at 3 h and starts to decline at 10 h, in ATJ3, which express a truncated and apparently inactive ATM protein, these maximal levels of p53-Ser 15 phosphorylation are seen only 10 h after irradiation. Interestingly, however, in A(-T)5RM cells which express 70% of the normal ATM levels, the time-dependent phosphorylation changes parallel those of normal cells, but the total levels of p53-Ser15 phosphorylation remain lower at all time-points examined. These findings would thus suggest an association between defective G1 checkpoint and suboptimal phosphorylation of p53-Ser 15 during the early hours post DNA damage.

In conclusion, we have demonstrated that AT and A(-T) cells frequently express modest amounts of full-length (and only rarely truncated) ATM protein, despite the elevated frequency of truncation mutations of the ATM gene. When expressed, this full-length ATM protein may be intrinsically defectivel or quantitatively insufficient for the normal regulation of the p53-dependent G1 cell cycle checkpoint. We have also shown that AT heterozygotes express 50–60% of the normal ATM levels, but this deficit alone is insufficient to impair the G1 checkpoint. Other factors, including the type of wt/mut ATM mutations, could thus contribute to this defect.

### ACKNOWLEDGEMENTS

This work was financially supported by the italian Telethon grants E337 and E764, the Italian Association for Cancer Research (AIRC), by Grants-in Aid for Pediatric Research and Cancer

Research from the Ministry of Health and Welfare, Japan; by a comprehensive-10 year strategy for Cancer Control and grants of the Human Science Foundation and the Leukemia Research Fund, Japan. Silvia Panigone is supported by a FIRC fellowship.

### REFERENCES

Athma P, Rappaport R and Swift M (1996) Molecular genotyping shows that ataxiatelangiectasia heterozygotes are predisposed to breast cancer. *Cancer Genet Cytogenet* **92**: 130–134

Banin S, Moyal L, Shieh SY, Taya Y, Anderson CW, Chessa L, Smorodinsky NI, Prives C, Reiss Y, Shiloh Y and Ziv Y (1998) Enhanced phosphorylation of p53 by ATM in response to DNA damage. *Science* **281**: 1674–1677

Boder E (1985) Ataxia-telangiectasia: An overview. In *Ataxia-Telangiectasia: Genetics, Neuropathology and Immunology of a Degenerative Disease of Childhood* Gatti RA and Swift M (eds), pp. 1–63. Alan R. Liss, New York

Canman CE, Wolff AC, Chen CY, Fornace AJ and Kastan MB (1994) The p53 dependent G1 cell cycle checkpoint pathway and ataxia telangiectasia. *Cancer Res* **54**: 5404–5458

Canman CE, Lim DS, Cimprich KA, Taya Y, Tamai K, Sagakuchi K, Appella E, Kastan MB and Siliciano JD (1998) Activation of the ATM kinase by ionizing radiation and phosphorylation of p53. *Science* **281**: 1677–1679

Delia D, Greaves M, Villa S and DeBraud F (1984) Characterization of the response of human thymocytes and blood lymphocytes to the synergistic mitogenicity of 12-O-tetradecanoylphorbol-13-acetate (TPA)-ionomycin. *Eur J Immunol* **14**: 720–724

Delia D, Goi K, Mizutani S, Yamada T, Aiello A, Fontanella E, Lamorte G, Iwata S, Ishioka C, Krajewski S, Reed JC and Pierotti MA (1997) Dissociation between cell cycle arrest and apoptosis can occur in Li–Fraumeni cells heterozygous for p53 gene mutations. *Oncogene* **14**: 2137–2147

FitzGerald MG, Bean JM, Hedge SR, Unsal H, MacDonald DJ, Harkin DP, Finklestein DM, Isselbacher KJ and Haber DA (1997) Heterozygous ATM mutations do not contribute to early onset of breast cancer. *Nat Genet* **15**: 307–310

Gilad S, Khosravi R, Shkedy D, Uziel T, Ziv Y, Savitsky K, Rotman G, Smith S, Chessa L, Jorgenson TJ, Harnik R, Frydman M, Sanal O, Portnoi S, Goldwicz Z, Jaspers NGJ, Gatti RA, Lenoir G, Lavin MF, Tatsumi K, Wegner RD, Shiloh Y and Bar-Shira A (1996) Predominance of null mutations in ataxia-telangiectasia. *Hum Mol Genet* **5**: 433–439

Gilad S, Chessa L, Khosravi R, Russel P, Galanty Y, Piane M, Gatti RA, Jorgensen TJ, Shiloh Y and Bar-Shira A (1998*a*) Genotype-phenotype relationships in Ataxia-Telangiectasia (A-T) and A-T variants. *Am J Hum Genet* **62**: 551–561

Gilad S, Khosravi R, Harnik R, Ziv Y, Shkedy D, Galanty Y, Frydman M, Levi J, Sanal O, Chessa L, Smeets D, Shiloh Y and Bar-Shira A (1998*b*) Identification of ATM mutations using extended RT-PCR and restriction endonuclease fingerprinting, and elucidation of the repertoire of A-*T* mutations in Israel. *Hum Mutat* **11**: 69–75

Hoekstra MF (1997) Responses to DNA damage and regulation of cell cycle checkpoints by the ATM protein kinase family. *Curr Opin Genet Dev* **7**: 170–175

Janin N, Andrieu N, Ossian K, Laugé A, Croquette MF, Debré M, Bressac de Paillerets B, Aurias A and Stoppa-Lyonnet D (1999) Breast cancer risk in ataxia-telangiectasia (AT) heterozygotes: haplotype study in French AT families. *Br J Cancer* **80**: 1042–1045

Khanna KK, Beamish H, Yan J, Robson K, Williams R, Dunn I and Lavin MF (1995) Nature of G1/S cell cycle checkpoint in ataxia-telangiectasia. *Oncogene* **11**: 609–618

Lakin ND, Weber P, Stankovic T, Rottinghaus ST, Taylor MAR and Jackson SP (1996) Analysis of the ATM protein in wild type and ataxia telangiectasia cells. *Oncogene* **13**: 2707–2716

Lakin ND, Hann BC and Jackson SP (1999) The ataxia-telangiectasia related protein ATR mediates DNA-dependent phosphorylation of p53. *Oncogene* **18**: 3989–3995

Meyn SM (1995) Ataxia-telangiectasia and cellular responses to DNA damage. *Cancer Res* **55**: 5991–6001

Morgan SE, Lovly C, Pandita TK, Shiloh Y and Kastan MB (1997) Fragments of ATM which have dominant negative or complementing activity. *Mol Cell Biol* **17**: 2020–2029

Nakagawa K, Taya Y, Katsuyuki T and Yamaizumi M (1999) Requirement of ATM in phosphorylation of the human p53 protein at serine 15 following DNA double-strand breaks. *Mol Cell Biol* **19**: 2828–2834

Prudente S, Piane M, Brusco A, Saviozzi S, Carbonara A and Chessa L (1999) Mutations of the ATM gene in Italy recovered through different methodological approaches. *Bull Cancer*, in press.

Sanchez Y, Wong C, Thoma RS, Richman R, Wu Z, Piwnica-Worms H and Elledge SJ (1997) Conservation of the Chk1 checkpoint pathway in mammals: linkage of DNA damage to Cdk regulation through Cdc25. *Science* **277**: 1497–1501

Savitsky K, Bar-Shira A, Gilad S, Rotman G, Ziv Y, Vanagaite L, Tagle DA, Smith S, Uziel T, Sfez S, Ashkenazi M, Pecker I, Frydman M, Harnik R, Tatanjali SR, Simmons A, Clines GA, Sartiel A, Gatti RA, Chessa L, Sanal O, Lavin MF, Jaspers NGJ, Taylor MAR, Arlett CF, Miki T, Weissman SM, Lovett M, Collins FS and Shiloh Y (1995*a*). A single ataxia telangiectasia gene with a product similar to Pl-3 kinase. *Science* **268**: 1749–1753

Savitsky K, Sfez S, Tagle D, Ziv Y, Sartiel A, Collins FS, Shiloh Y and Rotman G (1995*b*) The complete sequence of the coding region of the ATM gene reveales similarity to cell cycle regulators in different species. *Hum Mol Genet* **4**: 2025–2032

Scott D, Hu Q and Roberts SA (1996) Dose-rate sparing for micronucleus induction in lymphocytes of controls and ataxia-telangiectasia heterozygotes exposed to 60Co γ- irradiation in vitro). *Int J Radiat Biol* **70**: 521–527

Shieh SY, Ikeda M, Taya Y and Prives C (1997) DNA damage-induced phosphorylation of p53 alleviates inhibition by MDM2. *Cell* **91**: 325–334

Shigeta T, Takagi M, Delia D, Chessa L, Iwata S, Kanke Y, Asada M, Eguchi M and Mizutani S (1999) Defective control of apoptosis and mitotic spindle checkpoint in heterozygous carriers of ATM mutations. *Cancer Res* **59**: 2602–2607

Siliciano JD, Canman CE, Taya Y, Sakaguchi K, Appella E and Kastan MB (1997) DNA damage induces phosphorylation of the amino terminus of p53. *Genes Dev* **11**: 3471–3481

Stilgenbauer S, Schaffner C, Litterst A, Liebisch P, Gilad S, Bar-Shira A, James MR, Lichter P and Dohner H (1997) Biallelic mutations in the ATM gene in T-prolymphocytic leukemia. *Nat Med* **3**: 1155–1159

Swift M, Morrel D, Cromartie E, Chamberlin R, Skolnick MH and Bishop DT (1986) The incidence and gene frequency of ataxia-telangiectasia in the United states. *Am J Hum Genet* **39**: 573–583

Swift M, Morrell D, Massey RB and Chase CL (1991) Incidence of cancer in 161 families affected by Ataxia-Telangiectasia. *New Engl J Med* **325**: 1831–1836

Takagi M, Delia D, Chessa L, Iwata S, Shigeta T, Kanke Y, Goi K, Asada M, Eguchi M, Kodama C and Mizutani S (1998) Defective control of apoptosis, radiosensitivity, and spindle checkpoint in ataxia-telangiectasia. *Cancer Res* **58**: 4923–4929

Tchirkov A, Bay JO, Pernin D, Bignon YJ, Rio P, Grancho M, Kwiatkowski F, Giollant M, Malet P and Verrelle P (1997) Detection of heterozygous carriers of the ataxia-telangiectasia (ATM) gene by G2 phase chromosomal radiosensitivity of peripheral blood lymphocytes. *Hum Genet* **101**: 312–316

Teraoka SN, Telatar M, Becker-Catania S, Liang T, Onengut S, Tolun A, Chessa L, Sanal O, Bernatowska E, Gatti RA and Concannon P. Splicing defects in the ataxia-telangiectasia gene, ATM: underlying mutations and consequences (1999) *Am J Hum Genet* **64**: 1617–1631

Thacker J (1994) Cellular radiosensitivity in ataxia-telangiectasia. *Int J Radiat Biol* **66**: S87–S96

Tibbetts RS, Brumbaugh KM, Williams JM, Sarkaria JN, Cliby WA, Shieh SY, Taya Y, Prives C and Abraham RT (1999) A role for ATR in the DNA damage-induced phosphorylation of p53. *Genes Dev* **13**: 152–157

Vorechovsky I, Rasio D, Luo L, Monaco C, Hammarstrom L, Webster ADB, Zaloudij J, Barbanti-Brodano G, James M, Russo G, Croce CM and Negrini M (1996) The ATM gene and susceptibility to breast cancer: analysis of 38 breast tumors reveal no evidence for mutations. *Cancer Res* **56**: 2726–2732

Vorechovsky I, Luo L, Dyer MJS, Catovsky D, Amlot PL, Yaxley JC, Foroni L, Hammarstrom L, Websrter ADB and Yuille MAR (1997) Clustering of missense mutations in the ataxia telangiectasia gene in a sporadic T-cell leukemia. *Nat Genet* **17**: 96–99

Walworth NC and Bernards R (1997) Rad-dependent response of the chk1-encoded protein kinase at the DNA damage checkpoint. *Science* **271**: 353–356

Watters D, KK Khanna, Beamish H, Birrel G, Spring K, Kedar P, Gatei K, Stenzel D, Hobson K, Kozlov S, Zhang N, Farrel A, Ramsay J, Gatti R and Lavin M (1997) Cellular localization of the ataxia-telangiectasia (ATM)gene product and discrimination between mutated and normal form. *Oncogene* **14**: 1911–1921

West CML, Elyan SAG, Berry P, Cowan R and Scott D (1995) A comparison of the radiosensitivty of lymphocytes from normal donors, cancer patients individuals with ataxia-telangiectasia (A-T) and AT heterozygotes. *Int J Radiat. Biol* **68**: 197–203

Westphal CH, Schmaltz C, Rowan S, Elson A, Fisher DE and Leder P (1997) Genetic interaction between atm and p53 influence cellular proliferation and irradiation-induced cell cycle checkpoints. *Cancer Res* **57**: 1664–1667

 *© 2000 Cancer Research Campaign*

Wright J, Teraoka S, Onegut S, Tolun A, Gatti RA, Ochs HD and Concannon P (1996) A high frequency of distinct ATM mutations in ataxia-telangiectasia. *Am J Hum Genet* **59**: 839–846

Zhang N, Chen P, KK Khanna, Scott S, Gatei M, Kozlov S, Watters D, Spring K, Yen T and Lavin MF (1997) Isolation of full length ATM cDNA and correction of the ataxia-telangiectasia cellular phenotype. *Proc Natl Acad Sci USA* **94**: 8021–8026

Ziv Y, Bar-Shir A, Pecker I, Russel P, Jorgesen TJ, Tsarfati I and Shiloh Y (1997) Recombinant ATM protein complements the cellular A-T phenotype. *Oncogene* **15**: 159–157

© 2000 Cancer Research Campaign
